# Presence of *Phlebotomus perniciosus* Atypical Form in Algeria

**Published:** 2017-03-14

**Authors:** Kamel Eddine Benallal, Razika Benikhlef, Rafik Garni, Brahim Gassen, Jean-Pierre Dedet, Zoubir Harrat

**Affiliations:** 1 Laboratoire d’Eco-Epidémiologie Parasitaire et Génétique des Populations, Institut Pasteur d’Algérie, Algeria; 2 Direction de la Santé Publique de Tamanrasset, Algeria; 3 Université Montpellier 1 et CHRU de Montpellier, Centre National de Référence des Leishmanioses, Département de Parasitologie-Mycologie, France

**Keywords:** *Phlebotomus perniciosus*, *Phlebotomus longicuspis*, Atypical *phlebotomus perniciosus*, Leishmaniasis, Algeria

## Abstract

**Background:**

*Phlebotomus perniciosus* and *Phlebotomus longicuspis* are two phlebotomine sand fly species morphologically similar and differing in males only by the shape of the copulatory valves which are bifurcated in *P. perniciosus*, tip long and tapered in *P. longicuspis*.

**Methods:**

A count of the median coxite setae was carried out on 208 specimens from the collections of Dedet and of Parrot, identified previously as *P. longicuspis* and on 38 *P. perniciosus* male sand flies captured during the year 2012–2013, in order to seek the presence of atypical *P. perniciosus* form.

**Results:**

The analysis revealed the presence of 33/246 (13%) atypical *P. perniciosus* previously confused with *P. longicuspis* species and whose distribution is mainly located in the semi-arid and arid bioclimatic regions.

**Conclusion:**

This study proved for the first time the presence of atypical form of *P. perniciosus* in Algeria.

## Introduction

In Algeria, the first sand fly specimens were reported by Foley and Leduc (1912). Since then, several taxonomic studies were conducted on different species and how they might influence transmission of leishmaniasis in Algeria. The recent entomological surveys have reported the presence of *Phlebotomus* (*Transphlebotomus*) *mascittii* Grassi, 1908, *P.* (*Larroussius*) *chadlii* females in the northeast and *P.* (*Paraphlebotomus*) *kazeruni* in the extreme south of Algeria (Berdane-Brouk et al. 2011, Benallal et al. 2013). In the Mediterranean basin, *Phlebotomus* (*Larroussius*) *perniciosus* Newstead, 1911 is the most common phlebotomine sand fly, related to humid and arid bioclimatic stages. Its distribution spreads out up to the Saharan edge of Tassili and Hoggar, with higher abundance in the humid areas (Rioux et al. 1967, Dedet et al. 1984, Berdjane-Brouk et al. 2012).

**Fig. 1.**
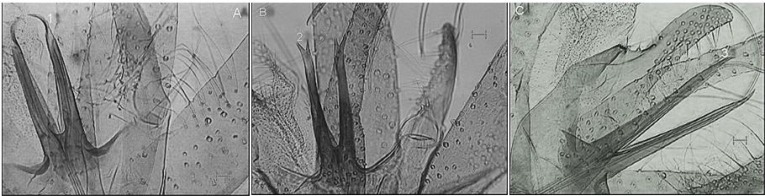
Copulatory valves of *Phlebotomus perniciosus* and *Phlebotomus longicuspis*. A) atypical form of *Phlebotomus perniciosus*, B) typical form of *Phlebotomus perniciosus*, C) typical form of *Phlebotomus longicuspis*, 1) curved form of copulatory valves, 2) forked form and 3) slightly curved, single point. Bar = 10 μm

In Algeria, it is the main vector of *Leishmania infantum* MON-1, the parasite that causes the majority of the visceral leishmaniasis (VL) cases in humans and dogs (Izri et al. 1990). This species is often collected in sympatry with a closely related species, *Ph.* (*Larroussius*) *longicuspis* Nitzulescu, 1930. Parrot considered *P. longicuspis* as a potential vector of *L. infantum* in the arid areas (Parrot et al. 1941) and recently, this species was found naturally infected with this parasite (Berdjane-Brouk et al. 2012). Females of the *Larroussius* subgenus are differentiated mainly by the aspect and the dilatation of the distal spermathecal ducts (Léger et al. 1983, Killick-Kendrick et al. 1990). However, males are distinguished by the morphology of the copulatory valves (aedeagus), with bifurcated apex in *P. perniciosus* (PN) (Fig. 1B) and with a long, slightly curved, single pointed apex in *P. longicuspis* (LC) (Fig. 1C) (Parrot 1936 a and b).

Previous studies on *P. perniciosus* males based on the number of median coxite setae highlighted atypical forms of *P. perniciosus* (PNA) confused with *P. longicuspis* mainly in Spain (Morrillas-Marquez et al. 1991, Collantes and Martinez-Ortega 1997, Martinez-Sanchez et al. 2000), in Morocco (Benabdennbi and Pesson 1998, Pesson et al. 2004, Guernaoui et al. 2006, Boussa et al. 2008) and recently in Tunisia (Ghrab et al. 2006, Boudabous et al. 2008). Then isoenzyme analysis has permitted distinguishing these morphs at hexokinase (HK) locus thus as molecular biology using random amplified polymorphic DNA (RADP) technique. The result of the three techniques has allowed including PNA with *P. perniciosus* species (Benabdennbi et al. 1999, Martinez-Sanchez et al. 2000, Pesson et al. 2004, Boudabous et al. 2012). Parrot and Durand-Delacre (1947) reported that a *P. longicuspis* males collected in BeniOunif (Oran Sahara) displayed a copulatory valves more curved than usual, and in 2012, we identified a male of the subgenus *Larroussius* collected in Tamanrasset (extreme south of Algeria) which exhibited the same features (curved copulatory valves with a single point).

This work aimed whether atypical forms of *P. perniciosus* in Algeria are present using the morphometric technique.

## Materials and Methods

In order to investigate the presence of atypical *P. perniciosus* males in Algeria, a retrospective study was done on 208 specimens previously identified as *P. longicuspis* belonging to the two collections, of Louis Parrot (specimens collected between 1934 and 1958) and of Jean-Pierre Dedet (specimens collected between 1973 and 1975). In addition, other samples of the same species (*P. perniciosus* and *P. longicuspis*) were captured in animal shelters between 2012 and 2013 by sticky traps in different localities of the country (Setif, Oum-Bouaghi, Blida, Ghardaïa and Tamanrasset) and included in the study.

Only males of *P. perniciosus* and *P. longicuspis* were slide mounted with Canada balsam after treatment in NaOH 20% (Abonnenc 1972). In all cases, examination of copulatory valves morphology following the key of Dedet (1984) was associated to a counting of the median coxite setae using a Motic 210 camera. The details of the examined specimens and their place of trapping according to bioclimatic zones are summarized in (Table 1).

**Table 1.** Sand flies origin and numbers of specimens studied, according to bioclimatic stages

**Bioclimatic zone**	**Region**	**(n)**	**Collection site GPS coordinate**
	Tizi Ouzou*	30	36°32′20.88″N 4°26′28.55″E
**Humid**	Blida ^(C)^	4	36°33′22.45″N 3° 3′17.06″E
	Blida**	7	/
	Béjaïa*	7	36°37′40.21″N 5°20′42.62″E
**Subhumid**	Boumerdes*	5	36°43′47.24″N 3°32′17.61″E
	Alger**	35	/
	Sétif ^(C)^	26	35°41′27.74″N 5°25′43.75″E
**Arid**	El Bayadh*	1	33°10′3.76″N 0°28′27.47″E
	Naâma*	6	32°29′31.70″N 0°28′4.65″O
	Ain Defla*	1	36°15′47.17″N 2°12′39.07″E
	Bouira*	3	36° 8′47.98″N 3°49′58.83″E
	Oran*	7	35°41′54.90″N 0°38′23.72″O
	Oum Bouaghi^(C)^	6	35°52′30.79″N 7° 6′48.95″E
**Semi-arid**	Relizane*	3	35°42′59.07″N 0°45′21.03″E
	Sidi-Bel-Abbes*	3	35°14′25.07″N 0°14′43.24″O
	Tlemcen*	22	34°38′27.30″N 1°33′41.14″O
	Tlemcen**	34	/
	Batna**	20	/
	Constantine**	1	/
	Béchar*	1	31°55′33.51″N 1°50′19.16″O
	Béchar**	7	/
	Ghardaïa ^(C)^	1	32°28′55.09″N 3°42′3.59″E
**Saharan**	Tamanrasset ^(C)^	1	22°53′23.07″N 5°21′33.07″E
	Tamanrasset**	5	/
	Biskra*	4	35°10′48.41″N 6° 0′32.26″E
	Biskra**	6	/
**Total**		246	

(*) Collection of Dedet,

(**) Collection of Parrot,

(C) Collected,

(/) Data not available and (*n*) Sample size.

Morphometric data (number of median coxite setae) were compared using an analysis of variance with *t* test of Student with Excel software version 2007.

## Results

**Fig. 2.**
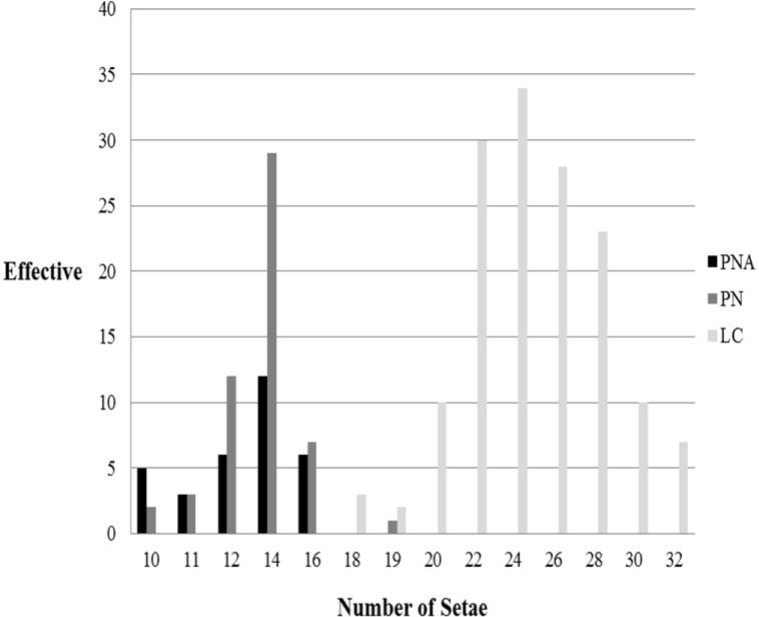
Frequency of median coxite setae for *Phlebotomus perniciosus* atypical (PNA), *Phlebotomus perniciosus* (PN) and (LC) *Phlebotomus longicuspis*.

**Fig. 3.**
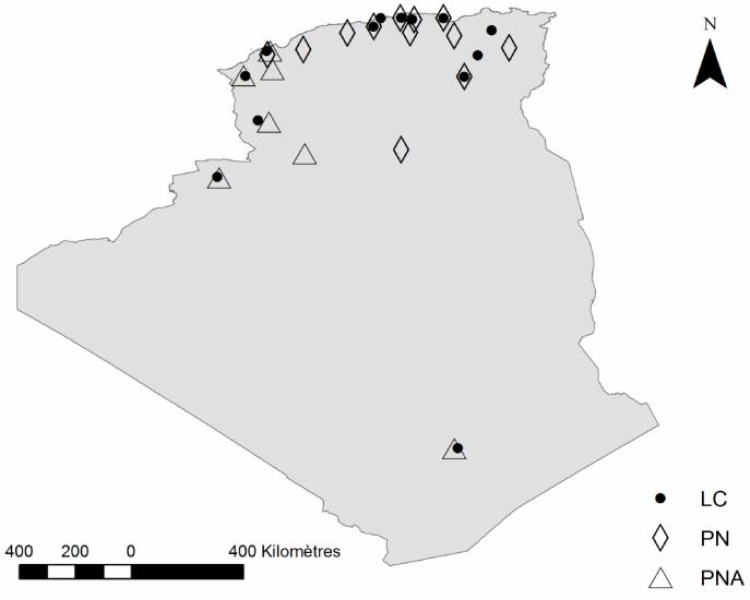
Distribution map of *Phlebotomus perniciosus* and *Phlebotomus longicuspis* in Algeria. LC: *P. longicuspis*, PN: *P. perniciosus*, PNA: atypical *P. perniciosus*.

Overall, 246 males belonging to *P. perniciosus* and *P. longicuspis* were analyzed. Within the 208 males of the two collections previously identified as *P. longicuspis* and 38 collected in field, 33 sand flies presented curved copulatory valves characteristic of *P. perniciosus* atypical male (Fig. 1A). The counted setae number of these males varied between 10 and 16 setae (Fig. 2). The number of setae allowed classifying the atypical males with *P. perniciosus* species that possessed between 10 and 19 coxite setae (Fig. 2) and forked copulatory valves (Fig. 1B). However, the number of median coxite setae of *P. longicuspis* varied between 18 and 32 (Fig. 2) with long, slightly curved, single point (Fig. 1C). The number of specimens examined and their setae mean numbers (+/− Standard Deviation) are summarized in (Table 2) with the minimal and maximal numbers of setae. The Student’s *t*-test showed significant differentiation between *P. perniciosus* and *P. longicuspis* populations (P< 0.05) (Table 2). The distribution of PNA form is very marked in northwest part of Algeria (Tlemcen and Sidi Bel-Abbes districts) and remained scarce in the rest of regions whereas the abundance of *P. perniciosus* decreased once moved southward over the country (Fig. 3).

**Table 2.** Comparison of the average numbers of median coxite setae by Student test “*t”* of *Phlebotomus perniciosus* and *Phlebotomus longicuspis*

**Species**	**Number of measure**	**Setae number**	**Min**	**Max**
***P. perniciosus***	96	13.67±3.39	10	18
***P. longicuspis***	150	24.33±5	18	32
**Student’ s test**	20.69	ddl= 13	P= 0.000	1385

## Discussion

The two collections conserved in Pasteur Institute of Algeria, collection of Parrot which counts 114 species distributed over 16,482 slides, most species belong to the African continent (164 species from 16 countries), to the European continent (36 species from 08 countries) and to the Middle East and Asia (29 species from nine countries), and that of Dedet which counts 4,689 slides with 15 species originating only from Algeria.

Despite the small number of specimens reviewed due to the improper mounting of some specimens which did not allow performing a correct counting of the median coxite setae our results provide, for the first time, the evidence for atypical forms of *P. perniciosus* in Algeria and thus correct the identification of several specimens identified as *P*. *longicuspis*. The number of median coxite setae remains a powerful tool in the morphological identification since it was previously used to distinguish between *P. ariasi/P. chadlii* and *P. neglectus/P. syriacus* of *Larroussius* subgenus (Benabdennbi and Pesson 1998). It has allowed the description of *P. perniciosus* atypical male form in Morocco, Spain and Tunisia (Morrillas-Marquez et al. 1991, Benabdennbi and Pesson 1998, Ghrab et al. 2006). The bioclimatic and geographic repartition of the different analysed sand flies highlighted a different distribution between PN and PNA forms. However, PN are more abundant in the north and northeast of Algeria. This form occupies humid and sub humid bioclimatic zones in concordance with the previous entomological surveys conducted in Mila and Jijel by Berchi et al. (2007). Nevertheless the abundance of PNA is rather from the northwest to the great south, thus occupying semi arid, arid and Saharan bioclimatic zones.

In Morocco, PNA repartition spreads out from northeast to the south at the Morocco-Algerian coast (Boussa et al. 2008), whereas in Tunisia its distribution is higher to south in the arid and semi arid areas (Boudabous et al. 2012). Consequently, in Maghreb the repartition of PNA obeys to the same bioclimatic and geographic conditions and seems to be linked to arid climate. Further investigations in Libya where distribution of *P. perniciosus* is limited in North Africa (Benabdennbi and Pesson 1998) should give more details about *P. perniciosus* morphs distribution. The repartition of *P. longicuspis* is large and this species is found from the north up to Tamanrasset in the extreme south of Algeria. Recently, its distribution area has won more ground since it has been reported also in Burkina Faso (Depaquit et al. 2005). Nonetheless, LC density remains very low comparing to *P. perniciosus* and it is found in humid to Saharan bioclimatic stages (Dedet et al. 1984).

## Conclusion

Association of median coxite setae to the standard morphological criteria revealed the presence of 33/246 (13%) of atypical *P. perniciosus* until now confused with *P. longicuspis* species mainly located in the semi arid and arid areas. The morphometric tool allowed us to draw a new distribution map of PN, PNA and LC in Algeria and to add the atypical form of *P. perniciosus* into the list of Algerian phlebotomine fauna. Further biochemical, molecular and morphometric studies should be done in order to highlight the status of the different *P. perniciosus* morphs of Algeria, and also it is important to check the competence of PNA females to develop and to transmit *Leishmania* sp parasite.
